# Plantar Pressure Detection System Based on Flexible Hydrogel Sensor Array and WT-RF

**DOI:** 10.3390/s21175964

**Published:** 2021-09-06

**Authors:** Wei Liu, Yineng Xiao, Xiaoming Wang, Fangming Deng

**Affiliations:** School of Electrical and Automation Engineering, East China Jiaotong University, Nanchang 330013, China; 8502@ecjtu.edu.cn (W.L.); 2019028080800015@ecjtu.edu.cn (Y.X.); 2501@ecjtu.edu.cn (X.W.)

**Keywords:** plantar pressure detection, gait recognition, flexible hydrogel sensor

## Abstract

This paper presents a hydrogel-based flexible sensor array to detect plantar pressure distribution and recognize the gait patterns to assist those who suffer from gait disorders to rehabilitate better. The traditional pressure detection array is composed of rigid metal sensors, which have poor biocompatibility and expensive manufacturing costs. To solve the above problems, we have designed and fabricated a novel flexible sensor array based on AAM/NaCl (Acrylamide/Sodium chloride) hydrogel and PI (Polyimide) membrane. The proposed array exhibits excellent structural flexibility (209 KPa) and high sensitivity (12.3 mV·N^−1^), which allows it to be in full contact with the sole of the foot to collect pressure signals accurately. The Wavelet Transform-Random Forest (WT-RF) algorithm is introduced to recognize the gaits based on the plantar pressure signals. Wavelet transform realizes the signal filtering and normalization, and random forest is responsible for the classification of the processed signals. The classification accuracy of the WT-RF algorithm reaches 91.9%, which ensures the precise recognition of gaits.

## 1. Introduction

Patients with lower limb diseases or neurological diseases often have problems such as difficulty walking and chaotic steps [1]. In order to help this group of people carry out rehabilitation walking training, it is necessary to design a plantar pressure detection system. In this way, doctors can formulate specific rehabilitation plans for them based on the changes in the patient’s gait during walking.

To realize gait recognition, it is crucial to gather plantar pressure signals. The pressure detection array has been studied by many scholars [2,3,4], and its material types can be classified into rigid metal and flexible membranes. A three-layer pressure-sensing array is proposed in [5]. The middle layer of the array is made of piezo-resistive material, and the top and bottom layers are copper pads. The pressure-sensing array is used to analyze gait parameters after being cut into the shape of insole. Huang et al. used FSR (force-sensing resistor) as the sensing element to fabricate a kind of pressure sensor array. Combined with the triangle positioning algorithm, his team realized the detection of contact force and contact position. The results show that the force and space detection accuracy of the pressure sensor array reaches 88.23% [6].

However, the pressure-sensing arrays made of rigid metal materials show poor biocompatibility and ductility. As a consequence, it may cause allergic reactions or other adverse physiological impacts while in contact with human skin. The poor ductility greatly limits the application range of the pressure-sensing array. It cannot be applied in high-curvature or uneven surfaces. Sekitani et al. used PEN (polyethylene naphthalate) as a substrate and organic material as a conductive layer to fabricate a flexible pressure-sensing array. The fabricated array can detect the spatial distribution of applied mechanical pressure and convert it into two-dimensional images for storage [7]. So et al. embedded a PDMS layer in the vertical arrangement of carbon nanotubes to make a flexible pressure sensor, which realized the function of tactile sensing [8]. 

Classification of the collected pressure signals is the basis of gait recognition. Common classification algorithms include support vector machine (SVM) [9,10,11], artificial neural networks (ANN) [12,13,14], and random forests (RF) [15,16,17]. SVM is a binary classification model, which constructs a hyperplane with the largest geometric distance and maps it into a high-dimensional space to classify specific objects. In the experiment of using EMG to predict the angle of five knee joints, compared with classifiers, such as LDA and KNN, the classification accuracy of SVM is higher, reaching 93.07 ± 3.84% [18]. However, SVM classifier is inefficient when the number of samples is large, and it is difficult to find a proper kernel function for nonlinear classification problems [19]. M.F. et al. applied ANN to the classification of fatigue strain signal to obtain the best pattern recognition. The classification accuracy of ANN is 92%, and five levels of fatigue damage are obtained [20]. The ANN has a high demand for computing power and a long training time, so it is not suitable for the scene with real-time requirements. A random forest algorithm for EEG signal classification was exhibited in [21], and the accuracy reaches 89.9% after combining the common spatial pattern (CSP). The random forest algorithm is not ideal for data with few feature dimensions, so it is not applicable in the pressure signal classification of this article individually.

This paper proposes a flexible pressure-sensing array to achieve plantar pressure detection and gait recognition. Due to the flexibility and biocompatibility, the pressure-sensing array can be utilized well in plantar pressure detection. After obtaining the pressure signals, the Wavelet Transform-Random Forest (WT-RF) algorithm is applied in the preprocessing and classification of the gathered signals. Thus, different gaits can be recognized accordingly, and the comprehensive accuracy reaches 91.9%.

## 2. Materials and Methods

### 2.1. Design of Flexible Hydrogel Sensor Array

**Figure 1:**
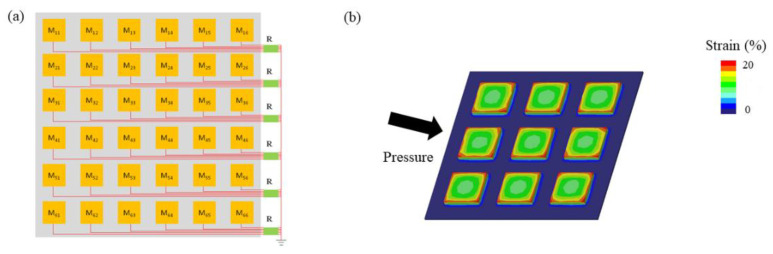
(**a**) Plane structure design of the FHSA. (**b**) Finite element analysis of the FHSA under pressure.

The flexible hydrogel sensor array (FHSA) is composed of three layers. The top and bottom layer sustain the overall structure and insulate electrical interference. The middle layer is the sensing layer, which is responsible for collecting pressure signals. The sensing layer contains 36 pressure-sensing units, which are arranged in a 66 matrix. Each row of sensor units output a total of six signals, which are connected to a bus through resistors. The overall design of FHSA’s structure is presented in Figure 1a. 

Ideally, the external force exerted on the FHSA can be decomposed into three forces: horizontal transverse ($F_{x}$), horizontal longitudinal ($F_{y}$), and vertical ($F_{z}$). The detection of pressure is based on the piezoresistive effect, and the change in relative resistance is presented in Equation (1),

(1) $$\frac{∆R}{R}=\pi_{x}F_{x}+\pi_{y}F_{y}$$

where $\pi_{x}$ and $\pi_{y}$ are the horizontal transverse and horizontal longitudinal piezoresistive coefficients. The $F_{x}$ and $F_{y}$ represent the force components in the corresponding direction. Moreover, based on the principle of elasticity, when a Z-axis pressure is applied to the FHSA, the strain force can be expressed as Equations (2) and (3).

(2) $$\sigma_{x}=\frac{2x^{2}}{\pi {\left(x^{2}+y^{2}\right)}^{2}}\left(xF_{x}+yF_{y}\right)$$

(3) $$\sigma_{y}=\frac{2y^{2}}{\pi {\left(x^{2}+y^{2}\right)}^{2}}\left(xF_{x}+yF_{y}\right)$$

When determining the dimension of FHSA, it is necessary to minimize the size under the premise of ensuring the accuracy of pressure detection. The accuracy is reflected in the relative change rate of resistance. Once the pressure range is determined, the optimal dimension of FHSA can be obtained according to Equation (4),

(4) $$\frac{∆R}{R}=\left(1+2v\right)\cdot \varepsilon +\frac{l}{w\cdot t}$$

where v is the Poisson rate, and ε is the value of pressure. The l, w, and t are the length, width, and thickness of the FHSA, respectively. After determining the optimal size of the FHSA, the strain force at any position on the FHSA is shown in Equation (5). By comparing the maximum strain force with the material strain threshold, the rationality of the designed structure can be evaluated.

(5) $$\sigma =\frac{3}{4\pi }\cdot \frac{t^{3}}{{\left(l^{2}w^{2}+t^{2}\right)}^{5/2}}F_{y}$$

Finite element analysis (FEA) plays a significant role in the structural design. We used software (Creo, Parametric Technology Corporation, Boston, MA, USA) to study the mechanical behavior of FHSA under pressure. The Young’s modulus (E) and Poisson ratio (v) of FHSA are measured and set at 209 KPa and 0.42. The degree of strain is analyzed to illustrate the mechanical stability of the FHSA, and the result of FEA is shown in Figure 1b.

### 2.2. Sensor Fabrication and Calibration

The top and bottom layers of FHSA are PI (Polyimide) films, and the material of middle layer is AAM/NaCl (Acrylamide/Sodium chloride).

The fabrication of hydrogel films can be achieved through the following steps. Add 7.82 g of AAM monomer powder (Aladdin Co., Shanghai, China) and 8.01 g of NaCl (Aladdin Co., Shanghai, China) to DI (deionized) water and keep stirring to dissolve them completely. After about 10 min, add 0.085 g of AP (Ammonium persulphate, Sigma-Aldrich Co., Saint Louis, MO, USA) and 0.03 g of MBAA (Methylene-Bis-Acrylamide, Sigma-Aldrich Co., Saint Louis, MO, USA) as a crosslinking agent to the mixed solution. Then, add 0.125 g of TEMED (Tetramethylethylenediamine) to improve the reaction rate and conductivity. Eventually, pour the solution into a glass mold and wait for it to form.

The PI film is prepared by the steps below. Coat PAA (polyamic acid) solution (Aladdin Co., Shanghai, China) on a clean glass flake evenly, and then transfer it to the oven. Adjust the temperature to 270 °C and continue heating for an hour. After natural cooling for 20 min, a piece of complete PI film can be peeled off from the glass flake.

Cut the prepared PI film and AAM/NaCl film into a 350 mm × 350 mm square and thirty-six squares (50 mm × 50 mm), respectively via laser cutting. Plasma cleaner is introduced to activate the surface of the above films. Twenty minutes later, take out the film, adhere them to each other, and place them on a heating plate to heat for half an hour at 70 °C to strengthen the adhesion. Then, connect the AAM/NaCl-based pressure-sensing units with copper wires and lead them out. Ultimately, encapsulate the FHSA with epoxy resin to improve its working stability.

Aiming at ensuring the accuracy of the pressure measurement, we have calibrated the fabricated FHSA. Six locations were randomly selected, and twelve different pressures were applied on them. We collected and calculated the average resistance and conductance of the FHSA under pressures, and determined the relationship between them and pressure. The results are presented in Table 1.

**Figure 2:**
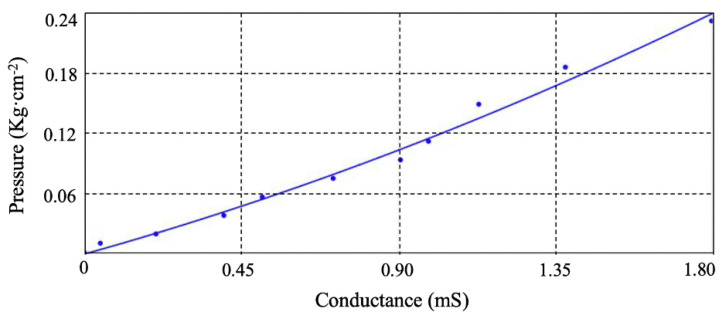
Curve fitting between the pressure applied on FHSA and its conductance.

Fitting the pressure value and FHSA’s conductance, the relational formula and fitting curve graph are shown in Equation (6) and Figure 2, respectively.

(6) $$p=0.022G^{2}+0.106G$$

### 2.3. Analysis and Classification of Pressure Signals

When the tester steps on the FHSA, the pressure exerted on different sensing units is unique. Consequently, these units show a distinct value of resistance. In order to collect the electrical signal changes of the FHSA when subjected to external pressure, we proposed the partial voltage method, whereby a constant voltage is applied to the FHSA through a power supply (SS-3020KD, Bufan Electronics Co., Ltd., Dongguan, China) to convert the resistance changes into voltage signals.

**Figure 3:**
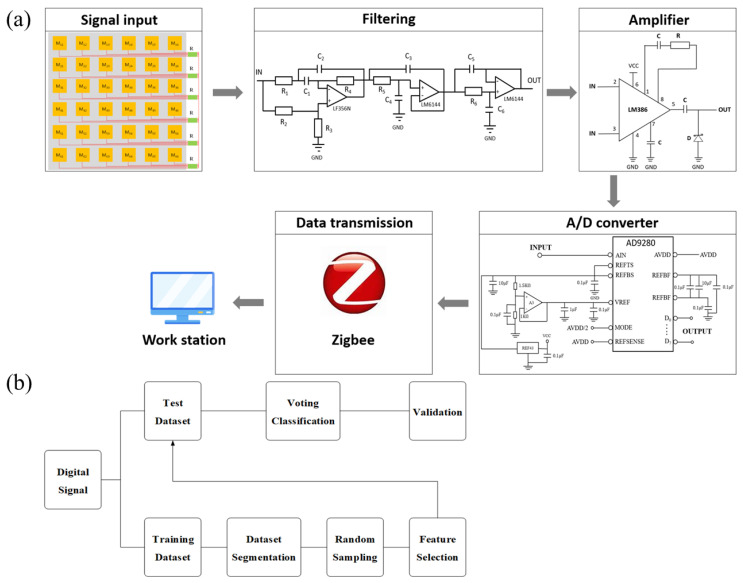
(**a**) Preprocessing of collected pressure signals. (**b**) Working flow of WT-RF algorithm.

The procedure of signal processing is presented in Figure 3a. The pressure signal output by the FHSA first passes through a filter circuit to filter out the high-frequency noise interference contained in the signal. Considering the weak strength of the voltage signal, we designed an amplifier to amplify it, which is conducive to subsequent processing and analysis. Before inputting to the computer via Zigbee, the signal is converted from an analog signal to digital form through an analog-to-digital conversion module.

In order to facilitate the subsequent data analysis, the maximum normalization method (Equation (7)) is introduced to process the voltage signals. In Equation (7), X represents the voltage signal amplitude at a certain moment, $X_{max}$ represents the maximum voltage value corresponding to the maximum pressure value of a single foot, and $X_{min}$ is the initial voltage. In this way, the pressure input is obtained and converted into a normalized voltage value. We propose a wavelet transform-random forest (WT-RF) algorithm (Figure 3b) to classify the input voltage signal to identify the corresponding gait. The wavelet transform algorithm realizes the denoising of the original signal and further improves the SNR (signal-to-noise ratio). The mathematical form of the original signal containing noise can be described as Equation (8).

(7) $$X_{Scale}=\frac{X-X_{min}}{X_{max}-X_{min}}$$

(8) f(t)=a(t)+ω·b(t)

Here, a(t) is the pure signal without noise, b(t) is the noise signal, and ω represents the intensity of noise. The difference in the characteristics of the pure signal and the noise signal in the wavelet transform leads to distinct coefficients obtained after the wavelet decomposition [22]. Using the low-frequency coefficients of wavelet decomposition and the high-frequency coefficients after threshold quantization to reconstruct, the denoised signal can be obtained.

Random forest is a feature classification method based on multiple decision trees, and its final classified result is decided by the voting of trees [23]. We collected the input voltage signals and randomly selected part of them as the original data set. Then, the original data set was divided into N training sets for training n decision trees. In the traditional random forest algorithm, each decision tree has the same weight in the voting decision process. In order to improve the final recognition accuracy, we evaluated different decision trees (Equation (9)) to give the better decision tree more weight,

(9) $$W\left(i\right)=1-\frac{\frac{1}{F\left(i\right)}}{\sum_{i=1}^{n}F\left(i\right)}-\frac{n-2}{n}$$

where W(i) is the weight of the *i*-th decision tree, n is the count of decision trees, and F(i) represents the comprehensive precision and recall of the *i*-th classifier. The algorithm features include signal amplitude, skewness, and kurtosis. The pressure signals corresponding to diverse gaits are distinct, and their signal features are also different, which is the basis of signal classification and gait recognition. We adjusted the main parameters (count of decision trees, maximum depth, and the minimum samples of leaf nodes) according to the difference in data training results. The optimization of above parameters helped us to achieve better classification accuracy and shorter training time.

## 3. Results

### 3.1. Performance Test

**Figure 4:**
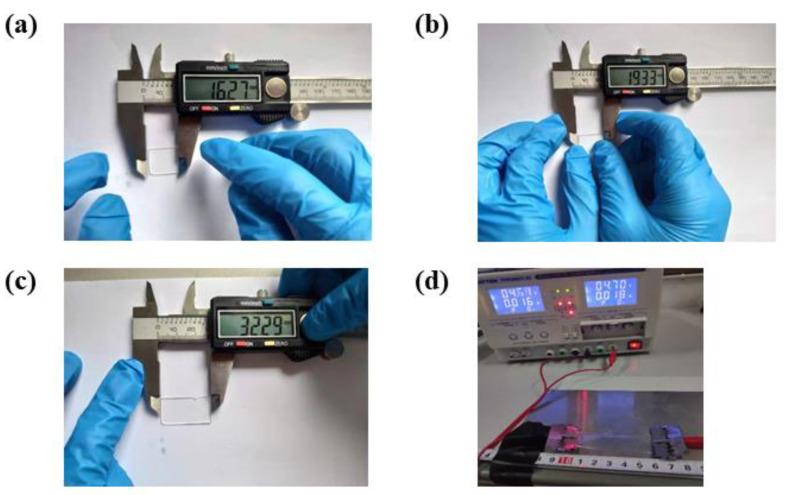
(**a**) Initial state. (**b**) During stretching. (**c**) Recovery after stretching. (**d**) Electrical performance test.

To illustrate the structural flexibility of the fabricated FHSA, we studied its mechanical properties by conducting the tensile and bending experiments (Figure 4a–c). The tensile test is realized on a programmable stretcher (IPBF-5, CARE Measurement & Control Co., Ltd., Tianjin, China). The FHSA is placed on the middle platform of the stretcher and clamped at both ends, and the stretching of it is realized by the movement of the clamps which are controlled by stepping motors. A digital multi-meter (MT-1820-C, Prokit’s Industries Co., Ltd., Taiwan, China) is connected to the FHSA to read the value of resistance (Figure 4d). The bending test was carried out on a bending test machine (YHS-216W-10kN-360, Yihua Instrument Technology Co., Ltd., Shanghai, China). One end of the FHSA is fixed, and the other end is connected with a clamp. The clamp moves along the track to make the FHSA bend between 0 and 180. Similarly, under different bending angles, the resistance of the FHSA is recorded by the digital multi-meter.

**Figure 5:**
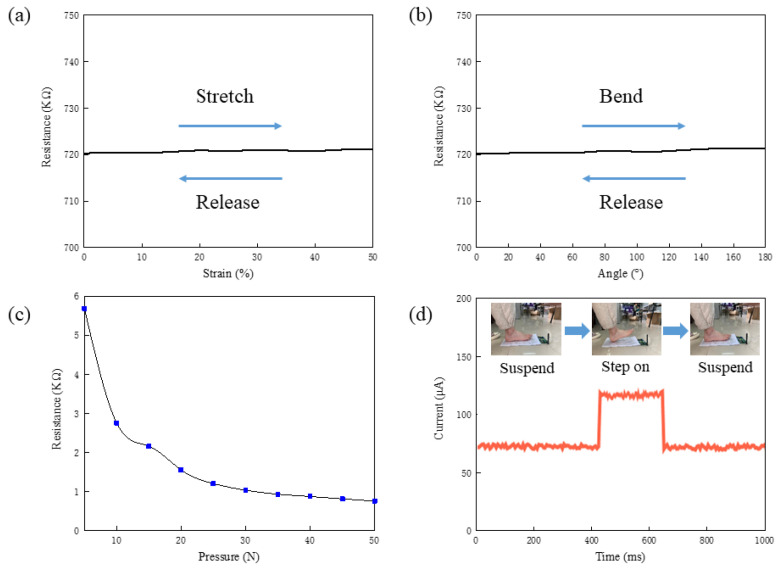
(**a**) Strain–resistance curve. (**b**) Angle–resistance curve. (**c**) The pressure acting on the FHSA and its resistance. (**d**) Response time of FHSA.

The relationship between resistance, material strain, and bending angle is shown in Figure 5a,b. In the process of repeatedly applying the tensile force to the material and releasing it, the resistance value of the material is measured in real time to quantify the structural flexibility, so as to ensure the stability and consistency of the structure under the action of external force. It can be seen from the results that when the material is elongated, its resistance value remains almost unchanged, which indicates that the designed FHSA can still work normally under 50% of the maximum strain. In the bending experiment, during bending and releasing, the resistance value of FHSA remains nearly the same. Therefore, the structure of FHSA can stay intact under the bending of 180° and can be applied in most bending scenes. 

When the FHSA is under pressure, its resistance value decreases. We applied different pressure to it and recorded the corresponding resistance value. The relationship between resistance and pressure is shown in Figure 5c. When the pressure is in the range of 0 N to 30 N, the resistance of the FHSA decreases obviously with the increase in pressure. This shows that the FHSA is more sensitive to the pressure change in a small range. To realize the accurate detection of high pressure, the increment in the count of pressure-sensing units is needed. Response time is an important parameter of the FHSA, which reflects the sensitivity to external pressure. Therefore, we applied a fixed voltage on the FHSA so as to measure its response time. After stabilizing, the pressure was exerted on the FHSA and the current curve was observed by an oscilloscope. The change curve of the current is shown in Figure 5d, and the response time of the FHSA is 69 ms.

### 3.2. Gait Recognition

The distribution of plantar pressure varies from person to person and may be affected by factors such as age, gender, and weight [24]. According to the formula of sampling quantity (Equation (10)), we recruited 30 volunteers and collected their plantar pressure distribution data,

(10) $$n=\frac{{\left(Z_{\frac{\alpha }{2}}\right)}^{2}P\left(1-P\right)}{E^{2}}$$

where n is the sample size, *Z* is the statistic, E is the sampling error, and P represents the sample proportion. We selected the commonly used confidence 90% and sample proportion 50%, and set the acceptable error to 15%. The values of the $Z_{\frac{\alpha }{2}}$, P, and E are 1.645, 0.5, and 0.15, respectively. After calculation, we finally determined the sample size as thirty. These volunteers are distributed in six age groups, half male and half female (Table 2). Based on these data, we examined the feasibility of the proposed FHSA and gait recognition algorithm and analyzed the effects of age, gender, and weight on the recognition of gaits. 

**Figure 6:**
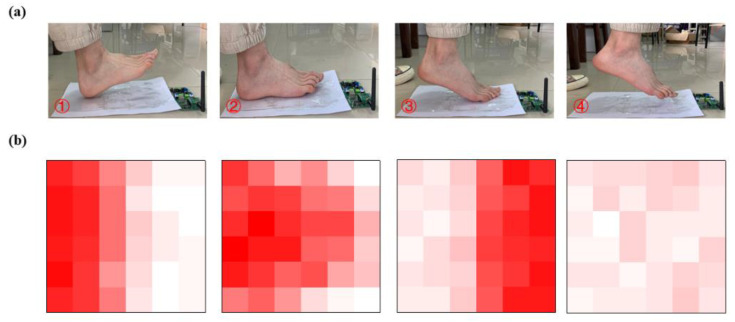
(**a**) Different gait patterns. (**b**) Two-dimensional pressure distribution map corresponding to different gaits.

The protocol for obtaining the pressure input is to have volunteers step on the FHSA placed on the ground and walk back and forth five times [25]. A total of six hundred sets of pressure data (left-foot and right-foot) of diverse gaits were gathered. We selected four different gait patterns (Figure 6a), and their pressure distributions are shown in Figure 6b.

**Figure 7:**
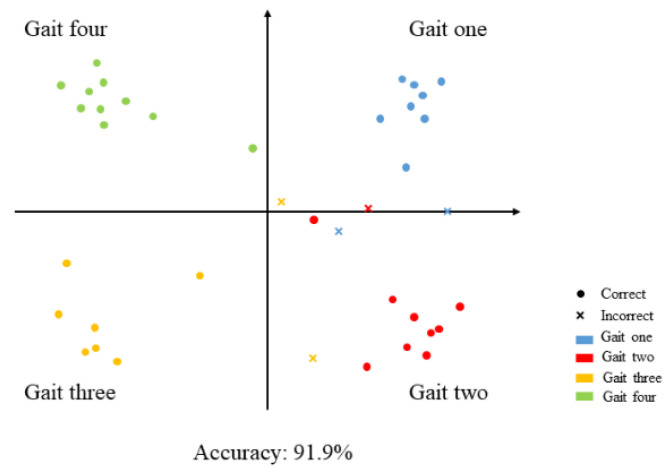
The classification result of diverse gait patterns.

We randomly selected 500 sets of data as the training set to train our proposed WT-RF algorithm model, and the remaining 100 sets of data were used for testing to obtain the gait recognition accuracy of the trained model. The classification results of the WT-RF algorithm are presented in Figure 7.

In order to determine the accuracy of the proposed WT-RF algorithm in pressure signal classification and gait recognition, we introduced four indicators (TP,FP,FN,TN) to comprehensively evaluate the classification results, where TP, FP, FN and TN refer to true positive, false positive, false negative, and true negative, respectively. Among them, true and false indicate whether the predicted gait is consistent with the actual gait, and true means that the classification is correct. Therefore, the classification and recognition accuracy of the WT-RF algorithm can be calculated by Equation (11), and the accuracy reaches 91.9%.

(11) $$\mathrm{Accuracy}=\frac{TP+TN}{TP+TN+FP+FN}$$

We introduced the Pearson correlation coefficient to analyze the influence of age, gender, and weight on the accuracy of gait recognition. The Pearson coefficient can be calculated by Equation (12), where X is age, gender, weight, and Y is the accuracy of gait recognition.

(12) $$\rho_{X,Y}=\frac{\sum \left(X-\bar{X}\right)\left(Y-\bar{Y}\right)}{\sqrt{\sum {\left(X-\bar{X}\right)}^{2}\sum {\left(Y-\bar{Y}\right)}^{2}}}$$

**Figure 8:**
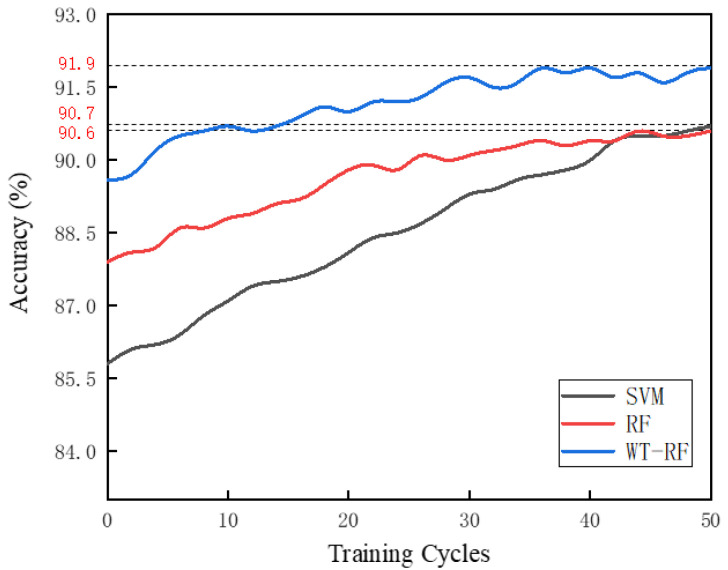
Comparison of accuracy of SVM (90.7%), RF (90.6%), and WT-RF algorithms (91.9%) under 50 training cycles.

After calculation, the Pearson coefficients of age, gender, and weight are 0.12, 0.09, and 0.18, respectively (in gender analysis, $X_{Female}=0,\;X_{Male}=1$). It can be seen that age and gender are not related to the accuracy of gait recognition, and the weight factor has a slightly higher correlation due to the optimal detection range of the FHSA. In addition, we trained the other two algorithms with the same data set and compared their classification accuracy with the WT-RF algorithm (Figure 8). Compared with the other two algorithms, WT-RF has higher classification accuracy even under few training cycles. When the training cycles are few, the classification accuracy of RF is significantly higher than that of SVM, but after 40 training cycles, their accuracies are very close. As a consequence, it is reasonable to select the WT-RF algorithm to classify the pressure signals and realize gait recognition.

At present, the mainstream plantar pressure detection system includes a platform system like this article and an in-shoe system [26]. An in-shoe device for monitoring plantar pressure is proposed in [27], which is composed of 64 pressure-sensitive elements. It can collect gait information at 100 Hz and draw a pressure curve to assess walking quality. Compared with the platform system, it has limitations on the spatial resolution of data and will affect the normal walking to a certain extent. Based on a gait database composed of 12 people, Kale et al. [28] introduced the view invariant method for gait recognition. The CCR (Correct Classification Rate) is 85%, which is significantly less than our work (91.9%).

## 4. Conclusions

This work combines flexible electronic technology and a signal classification algorithm to provide a new idea for plantar pressure detection and gait recognition. Compared with traditional rigid metal sensor arrays, the proposed FHSA with structural flexibility has higher precision of pressure detection and can be applied in more scenarios. The WT-RF algorithm is introduced to process and classify the pressure signals that gathered by FHSA to achieve gait recognition. The comprehensive accuracy of the proposed WT-RF algorithm reaches 91.9%, which is significantly higher than the traditional classification methods. The mode of flexible sensor array plus specific algorithms is suitable for many extended application scenarios, such as wind pressure detection and the wearable electronics field.

## Figures and Tables

**Table 1:** The relationship between the average resistance, conductance of the FHSA, and pressure.

Pressure (Kg·cm^−2^)	Resistance (KΩ)	Conductance (mS)
0	721.49	0
0.01	21.34	0.05
0.02	4.87	0.21
0.04	2.51	0.40
0.06	1.97	0.51
0.08	1.41	0.71
0.1	1.11	0.9
0.12	1.02	0.98
0.16	0.89	1.12
0.2	0.73	1.37
0.25	0.56	1.79

**Table 2:** Specific information of the recruited volunteers.

Age Group	Age	Gender (Female/Male)	Weight (kg)	Left-Foot Pressure (kg)	Right-Foot Pressure (kg)
16–20	17	M	58.3	30.1	29.3
	18	M	60.5	31.6	29.9
	19	F	43.4	22.3	20.2
	20	F	50.6	26.8	25.4
	20	M	68.1	34.6	33.1
21–25	21	M	61.6	32.5	30.9
	23	F	47.8	24.5	22.8
	23	F	49.7	25.8	24.3
	24	M	52.2	27.6	25.1
	24	F	51	26	24.5
26–30	28	F	61.3	32.4	29.6
	28	M	74.3	37.6	36.8
	29	M	58.7	28.3	30.4
	29	M	83.9	43.1	41
	29	F	55.9	28.6	27.9
31–35	31	M	67.3	34.8	33.2
	31	M	75.2	36.5	39
	32	F	59.6	31.6	28.7
	33	F	48.7	25	23.9
	35	F	53.9	28.2	26
36–40	37	M	54.4	28.5	26.4
	39	F	46.1	23.7	22.3
	40	M	67.8	35.8	32.6
	40	M	73.5	36.9	35.7
	40	F	52	26.5	25.5
41–45	41	F	48.7	25.1	24.1
	42	F	59.3	31.4	28.2
	44	M	71.9	37	34.8
	44	F	55.2	27.9	26
	45	M	68	35.3	33.6

## Data Availability

Not applicable.
